# Bayesian Functional Data Clustering for Temporal Microarray Data

**DOI:** 10.1155/2008/231897

**Published:** 2008-04-17

**Authors:** Ping Ma, Wenxuan Zhong, Yang Feng, Jun S. Liu

**Affiliations:** ^1^Department of Statistics, University of Illinois, Champaign, IL 61820, USA; ^2^Department of Statistics, Harvard University, Cambridge, MA 02138, USA

## Abstract

We propose a Bayesian procedure to cluster temporal gene expression microarray profiles, based on a mixed-effect smoothing-spline model, and design a Gibbs sampler to sample from the desired posterior distribution. Our method can determine the cluster number automatically based on the Bayesian information criterion, and handle missing data easily. When applied to a microarray dataset on the budding yeast, our clustering algorithm provides biologically meaningful gene clusters according to a functional enrichment analysis.

## 1. INTRODUCTION

 Microarray technology enables the scientist to measure the mRNA expression levels of thousands of genes simultaneously. For a particular species of interest, one can make microarray measurements under many different conditions and for different types of cells (if it is a multicellular organism). Genes' expression profiles under these conditions often give the scientist some clues on biological roles of these genes. A group of genes with similar profiles are often “coregulated” or participants of the same biological functions.

When a series of microarray experiments are conducted sequentially during a biological process, we call the resulting dataset a “temporal” microarray dataset, which can provide insights on the underlying biology and help decipher the dynamic gene regulatory network. Clustering genes with similar temporal profiles is a crucial first step to reveal potential relationships among the genes.

Conventional clustering methods, such as the K-means and hierarchical clustering, do not take into consideration the correlation in the gene expression levels over time. Although it is possible to use a general multivariate Gaussian model to account for the correlation structure, such a model ignores the time order of the gene expressions. As evidenced in our example, the time factor is important in interpreting the results of gene expression clustering in temporal data. It is also possible to use an autoregression model to describe the gene expression time series, but such a model often requires stationarity, which is unlikely to hold in most temporal microarray data.

Recently, nonparametric analysis of data in the form of curves, that is, functional data, is subject to active research, see [1, 2] for a comprehensive treatment of functional data analysis; and curve-based functional clustering methods have emerged [3–7], but a rigorous assessment of the estimation precision is still lacking.

In this paper, we propose a Bayesian clustering method, which optimally combines the available information and provides a proper uncertainty measure for all estimated quantities. Our method is based on a mixture of mixed-effect smoothing splines models. For each cluster, we model its mean profile as a smoothing spline function and describe its individual gene's variation by a parametric random effect. Based on the theory of reproducing-kernel Hilbert spaces [8], we represent the mean expression curve as a linear combination of certain basis functions, which enables us to derive the full posterior distribution up to a normalizing constant. All the conditional distributions needed by a Gibbs sampler are also easy to compute and to sample from. Our method automatically takes care of the missing data and infers the number of clusters in the data. Using the method, we analyzed a microarray dataset of budding yeast, we found that the majority of the clusters we had obtained are enriched for known and expected biological functions.

Our method is not restricted to temporal microarray data, and can be applied to all curve clustering problems, especially for sparsely and irregularly sampled temporal data.

## 2. MATERIAL AND METHODS

### 2.1. Mixed-effect representation of gene expression profile

Let the expression value of the *i*th gene at time *t* be *y_it_*. To accommodate missing data that occasionally occurs in microarray experiment, we denote **t**_*i*_ = (*t*_1_,…, *t*_*n_i_*_) and **y**_*i*_ = (*y*_*i*1_,…, *y_in_i__*)^*T*^, where *n_i_* is the number of measurements of *i*th gene. Our mixed-effect smoothing spline model [9] for genes in one cluster is

(1) $$y_{i}=\mu (t_{i})+Z_{i}b_{i}+\epsilon_{i},$$

where ***μ***(**t**_*i*_) = (*μ*(*t*_1_),…*μ*(*t_ni_*))^*T*^ is the cluster's mean profile, **b**_*i*_ ∼ *N*(0, *B*) is the random effect to capture the intragene correlation, *Z_i_* is the known design matrix for the random effect, and *ϵ_i_* ∼ *N*(0, *σ* ^2^ *I*) is the random error independent of **b** and of each other.

**Figure 1:**
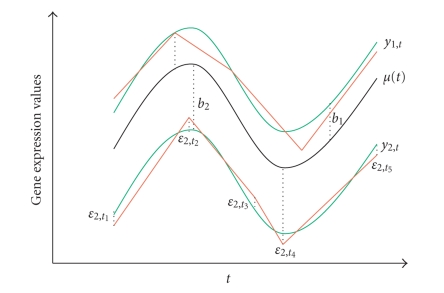
A smoothing-spline mixed effect model for temporal gene expression.

By taking different **b** vectors, we can accommodate different nonrandom effects. For example, when **b**_*i*_ = *b_i_* and *Z_i_* = **1**, the expression profile of the *i*th gene is parallel to the mean profile ***μ*** (Figure 1). If **b**_*i*_ = (*b*_*i*1_, *b*_*i*2_)^*T*^ and *Z_i_* = (**1**, **t**_*i*_), the difference between the *i*th gene profile and the mean profile is a linear function in time. More complicated structures such as periodicity can be modeled by letting the *Z_i_* be basis of a certain functional space.

By considering *μ* in a reproducing kernel Hilbert space *ℋ* ⊆ {*μ* : *M*(*μ*) < ∞} in which *M*(*μ*) is a square seminorm, we can represent *μ* as

(2) $$\mu (t)=\overset{m}{\underset{\nu =1}{\sum }}d_{\nu }\phi_{\nu }(t)+\overset{q}{\underset{i=1}{\sum }}c_{j}R_{M}(s_{j},t),\;t\in [0,a],$$

where {*s_j_*} is a set consisting of all distinct {*t_i_*}, *q* is the number of {*s_j_*}, and *R_M_* is the kernel of *ℋ*. The choice of *M*(*μ*) = ∫^*a*^_0_ (*d* ^2^ *μ*/*dt* ^2^)^2^ *dt* yields the cubic smoothing spline with

(3) $$\phi_{1}(t)=1,\;\;\phi_{2}(t)=t,$$

(4) $$R_{M}(t_{1},t_{2})=\int_{0}^{a}{\left(t_{1}-u\right)}_{+}{\left(t_{2}-u\right)}_{+}du,$$

where (·)_+_ = max(·, 0) [10].

Writing (2) in a vector-matrix form, we have

(5) $$\mu (t_{i})=S_{i}d_{i}+R_{i}c_{i},$$

where *S_i_* is *n_i_* × *m* with the (*i*, *ν*)th entry *ϕ*_*ν*_(*t_i_*) and *R* is *n_i_* × *q* with the (*i*, *j*)th entry *R_M_*(*t_i_*, *s_j_*). Substituting (5) into (1), we have

(6) $$y_{i}=S_{i}d_{i}+R_{i}c_{i}+Z_{i}b_{i}+\epsilon_{i}.$$

Denoting **y** = (**y** ^*T*^_1_,…, **y** ^*T*^_*n*_)^*T*^ and *S*, *R*, *Z*, ***ϵ*** similarly, we have the matrix representation

(7) y=Sd+Rc+Zb+ϵ,

where **b** = (**b** ^*T*^_1_,…, **b** ^*T*^_*n*_)^*T*^ ∼ *N*(0, diag(*B*,…, *B*)).

The prior distributions are specified as follows:

(8) $$\begin{array}{ll} d & ∼N(0,\text{diag}(\delta_{1},\ldots ,\;\delta_{m})),\\ c & ∼N(0,\;\tau^{2}I),\\ \sigma^{2} & ∼IG(\alpha_{\sigma^{2}},\;\beta_{\sigma^{2}}),\\ \tau^{2} & ∼IG(\alpha_{\tau^{2}},\;\beta_{\tau^{2}}),\\ B & ∼IW(\nu_{0},\;S_{0}^{-1}), \end{array}$$

where IG and IW are inverse-Gamma and inverse-Wishart distributions, respectively.

These priors lead to the following full conditional posteriors, which are used in our Gibbs sampler:

(9) $$\begin{array}{c} {}[d\;|\;b,\;c,\;\sigma^{2},\;\delta ,\;y]\;∼\;N(V_{d}S^{T}(y-Rc-Zb)/\sigma^{2},\;V_{d}),\\ {}[c\;|\;d,\;b,\;\sigma^{2},\;\tau^{2},\;y]\;∼\;N(V_{c}R^{T}(y-Sd-Zb)/\sigma^{2},\;V_{c}),\\ {}[b\;|\;d,\;c,\;\sigma^{2},\;B,\;y]\;∼\;N(V_{b}Z^{T}(y-Sd-Rc)/\sigma^{2},\;V_{b}),\\ {}[B\;|\;b]\;∼\;\text{I}\text{W}(\nu_{0}+n,{\left(S_{0}+\overset{n}{\underset{i=1}{\sum }}b_{i}b_{i}^{T}\right)}^{-1}),\\ {}[\tau^{2}\;|\;c]\;∼\;\text{I}\text{G}(\alpha_{\tau^{2}}+(q-m)/2,\beta_{\tau^{2}}+c^{T}c/2),\\ {}[\sigma^{2}\;|\;d,b,c,y]\;∼\;\text{I}\text{G}(\alpha_{\sigma^{2}}+n/2,\beta_{\sigma^{2}}+\text{SSR}), \end{array}$$

where *V_d_* = (*S^T^* *S*/ *σ* ^2^ + diag(*δ* ^−1^_1_,…, *δ* ^−1^_*m*_))^−1^, *V_b_* = (*Z^T^* *Z*/ *σ* ^2^ + diag(*B* ^−1^,…, *B* ^−1^))^−1^, *V_c_* = (*R^T^* *R*/ *σ* ^2^ + 1/*τ* ^2^ *I*)^−1^, and SSR = (**y** − *S* **d** − *R* **c** − *Z* **b**)^*T*^ (**y** − *S* **d** − *R* **c** − *Z* **b**).

### 2.2. The mixture model with unknown number of components

When more than one cluster is considered, we assume that the expression of the *i*th gene has a Gaussian mixture distribution:

(10) $$y_{i}∼p_{1}N(\mu_{1},\;\Sigma_{1})+\cdots +p_{K}N(\mu_{K},\;\Sigma_{K}),$$

where ***μ**_k_* and Σ_*k*_ = *ZB_k_Z^T^* + *σ* ^2^ *I* are the mean and covariance matrix for the *k*th component, as given by (7); *p*_*k*_ is the fraction of *k*th component, and *K* is the number of Gaussian components.

### 2.3. Class labels and cluster numbers

To ease the computation, we introduce a “latent” membership labeling variable *j_i_* for the *i*th gene so that

(11) $$y_{i}\;|\;J_{i}=j\;∼\;N(\mu_{j},\Sigma_{j}).$$

When the number of Gaussian components *K* is known, we can get the joint posterior probability as

(12) $$P(J,\mu ,\Sigma \;|\;y)=\pi (\mu ,\Sigma )\overset{n}{\underset{i=1}{\prod }}p_{j_{i}}N(y_{i}\;|\;\mu_{j_{i}},\Sigma_{j_{i}}),$$

where **J** = (*j*_1_,…, *j*_n_), ***μ*** = (*μ*_1_,…, *μ*_*K*_), Σ = (Σ_1_,…, Σ_K_), and *π*(***μ***, Σ) is the joint prior distribution.

Since *K* is unknown, we used the following Bayesian information criterion (BIC):

(13) BIC=−2log⁡p(y ∣ MK,θ^K)+lKlog⁡n,

where *M_K_* is the current model with parameters ***θ**_K_*, θ^K is the estimate, and *l_K_* is the total number of parameters in our model. A small BIC score indicates the adequacy of the corresponding model. An alternative to our current approach (i.e., each clustering configuration is equally likely given the number of clusters *K*, and *K* is determined by BIC) is to use a Polya Urn prior (also called the “Chinese restaurant” process), which postulates that when a new member comes in, its a priori probability for joining an existing cluster of size *m_i_* is (*m_i_* + *c*)/(*m* + *c*), and for forming a new cluster of its own is *c*/(*m* + *c*), where *m* is the total number of existing members. This prior, however, favors unbalanced cluster configurations (e.g., very large and very small clusters) and may not be appropriate in our applications.

#### 2.3.1. Gibbs Sampling from the Posterior

To complete our Bayesian analysis, we employ the Dirichlet prior Di (*α*_1_,…, *α*_*K*_) for (*p*_1_,…, *p*_*K*_), the cluster proportions. Thus, given the cluster indicator **J**, the posterior distribution of the *p*'s is again a Dirichlet distribution.

Given ***μ***_1_,…, ***μ***_*K*_, *B*_1_,…, *B*_*K*_, *σ* ^2^, we have the conditional distribution of *j_i_*:

(14) $$\begin{array}{ll} & p(J_{i}=j\;|\;\mu_{1},\ldots ,\mu_{K},B_{1},\ldots ,B_{K},\sigma^{2},y)\\ & \;\;=\frac{p_{j}N(y_{i}\;|\;\mu_{j},ZB_{j}Z^{T}+\sigma^{2}I)}{\sum_{k=1}^{K}p_{k}N(y_{i}\;|\;\mu_{k},ZB_{k}Z^{T}+\sigma^{2}I)}. \end{array}$$

With an initial value of **J**, which gives rise to a partition of **y** : (**y** ^*J*^_1_,…, **y** ^*J*^_*K*_), and the initial values of **d**_*k*_, **b**_*k*_, **c**_*k*_,*B_k_*, where *k* = 1,…, *K*, as well as *σ* ^2^, we iterate the following iterative conditional sampling steps:

- for *i* = 1,…, *n*, draw a new *j_i_* from the conditional distribution from (14) to replace the old one;
- conditional on **J**, sequentially
  - update **d**_*k*_ by a draw from [**d**_*k*_ | **b**_*k*_, **c**_*k*_, *σ* ^2^, ***δ***, **y** ^*J*^_k_], where *k* = 1,…, *K*,
  - update **b**_*k*_ from [**b**_*k*_ | **d**_*k*_, **c**_*k*_, *σ* ^2^, *B_k_*, **y** ^*J*^_*k*_], where *k* = 1,…, *K*,
  - update **c**_*k*_ from [**c**_*k*_ | **d**_*k*_, **b**_*k*_, *σ* ^2^, *τ* ^2^_*k*_, **y** ^*J*^_*k*_], where *k* = 1,…, *K*,
  - update *B_k_* ∼ [*B_k_* | **b**_*k*_], and *τ* ^2^_*k*_ ∼ [*τ* ^2^_*k*_ | **c**_*k*_], where *k* = 1,…, *K*,
  - update *σ* ^2^ ∼ [*σ* ^2^ | **d**, **b**, **c**, **y**],
  - update (*p*_1_,…, *p*_*K*_) ∼ Di(*n*_1_ + *α*_1_,…, *n_K_* + *α*_*K*_), where *n_j_* is the number of genes in the *j*th cluster.

## 3. RESULTS AND DISCUSSION

To study oxygen-responsive gene network, Lai et al. [11] used cDNA microarray to monitor the gene expression changes of wild-type budding yeast (*Saccharomyces cerevisiae*) under aerobic condition in galactose medium. Under aerobic condition, the oxygen concentration was lowered gradually until oxygen was exhausted during a period of ten minutes. Microarray experiments were conducted at 14 time points under aerobic condition. A reference sample was obtained from a pooled RNA collected from all time points for hybridization.

**Figure 2:**
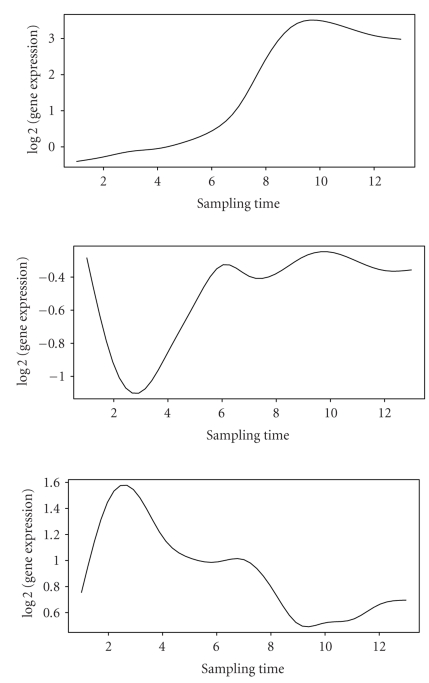
Estimated mean expression curves for cluster A, B, and C (from top to bottom) discovered in the yeast aerobic expression data.

For the analysis, Lai et al. [11] normalized gene expression after time 0 to gene expression of time 0 to set the common starting point. They identified 2388 genes whose expression is differentially expressed at one or more time points. Using our method, 2388 genes was clustered to 31 clusters, which yields the smallest BIC. FunSpec [12] was used for gene annotation and biological function enrichment analysis, where the Bonferroni-corrected functional enrichment *P*-values based on hypergeometric distributions are reported. We found 23 clusters out of 31 clusters discovered have biological functions over-represented. Among them, estimated mean gene expression profiles of three clusters are given in Figure 2.

In cluster A, which consists of 40 genes, the estimated mean expression goes up progressively as oxygen level goes down, which suggests that the genes in this cluster were transiently upregulated in response to aerobisis. Accordingly, genes involved in stress response (function enrichment *P*-value = 10^−4^) as well as cell rescue and defense are over-represented in this cluster (function enrichment *P*-value = 10^−4^). Furthermore, genes involved in molecular functions of oxidoreductase and coproporphyrinogen oxidase are also presented, which explains the upregulation of the gene expression levels.

We have 92 genes in cluster B, where the estimated mean gene expression drops down at the beginning rapidly and then goes up gradually. In this cluster, 34 genes are involved in protein synthesis (function enrichment *P*-value ≤ 10^−14^). Moreover, ribosome biogenesis are also over-represented (function enrichment *P*-value ≤ 10^−14^). These processes were affected by oxygen level initially, but were quickly adjusted to high expression levels to maintain living of yeast.

Contrast to cluster B, cluster C (68 genes) consists of genes involved in galactose fermentation (function enrichment *P*-value = 10^−4^), carbon utilization (functional enrichment *P*-value = 10^−2^), and carbohydrate metabolism (function enrichment *P*-value ≤ 10^−10^). The initial upregulation of gene expression under aerobic condition can be partly explained by the fact that the cell increases the energy uptaking through the carbon utilization as oxygen level goes down; but as the oxygen level continues to drop down, these processes are replaced by the more energy-efficient processes, which drives the expression levels of genes to be downregulated.

## 4. CONCLUSIONS

Conventional clustering methods do not take into consideration the correlation in the gene expression levels over time. Multivariate Gaussian models and time series analysis cannot model the time factor and correlation properly. These limitations can be readily overcome by the full Bayesian approach developed here. Although certain prior distributions and the related hyperparameters need to be input by the user, we found the clustering results rather robust to variations in such inputs. Moreover, our Bayesian clustering algorithm serves as a platform to incorporate more biological knowledge. Open source R code is available at www.stat.uiuc.edu/~pingma/BayesianFDAClust.htm.
